# Toxic Impacts of Trichlorfon on Tambaqui (*Colossoma macropomum*): Molecular Evidence of Oxidative, Metabolic and Apoptotic Stress

**DOI:** 10.3390/biology14121781

**Published:** 2025-12-13

**Authors:** Hallana Cristina Menezes da Silva, Daniele Aparecida Matoso, André Gentil da Silva, Ana Lúcia Silva Gomes, Wallice Paxiúba Duncan, Roberto Ferreira Artoni

**Affiliations:** 1 Laboratório de Genética e Evolução, Universidade Estadual de Ponta Grossa, Ponta Grossa 84010-330, PR, Brazil; 2 Laboratório de Biotecnologia e Citogenômica Animal, Departamento de Genética, Universidade Federal do Amazonas, Manaus 69067-005, AM, Brazil; danielematoso@yahoo.com.br; 3 Laboratório de Parasitologia de Animais Aquáticos, Graduação em Engenharia de Pesca, Universidade Federal do Amazonas, Manaus 69067-005, AM, Brazil; asswgentil@gmail.com; 4 Laboratório de Parasitologia de Animais Aquáticos, Departamento de Parasitologia, Universidade Federal do Amazonas, Manaus 69067-005, AM, Brazil; anapaimagomes@gmail.com; 5 Laboratório de Morfologia Funcional, Departamento de Morfologia, Universidade Federal do Amazonas, Manaus 69067-005, AM, Brazil; wduncan@ufam.edu.br; 6 Laboratório de Genética e Evolução, Departamento de Biologia Estrutural, Molecular e Genética, Universidade Estadual de Ponta Grossa, Ponta Grossa 84010-330, PR, Brazil; rfartoni@gmail.com

**Keywords:** Trichlorfon toxicity, *C. macropomum*, molecular biomarkers

## Abstract

Background: The intensification of aquaculture has led to increased use of chemical agents, such as trichlorfon, for controlling parasitic infections in farmed fish. While effective, this organophosphate compound may exert toxic effects even at sublethal concentrations, posing risks to economically important species such as tambaqui (*C. macropomum*). This study investigated the molecular effects of trichlorfon on the expression of genes involved in stress response, energy metabolism, and apoptosis in juvenile tambaqui. Methods: Fish were exposed to two sublethal concentrations of trichlorfon (30% and 50% LC_50–96 h_, equivalent to 0.261 and 0.435 mg/L) for 48, 72, and 96 h. Expression levels of *fkbp5*, *p53*, *pim-2*, *pir*, *me1*, *bbox1*, and *higd1a* were quantified in liver tissue using qPCR. Results: *fkbp5* and *p53* were strongly upregulated at 48 h, indicating acute stress and genotoxic activation. *me1* and *pim-2* were also upregulated, reflecting activation of compensatory energy metabolism and anti-apoptotic survival pathways. bbox1 showed an early induction followed by collapse at 96 h, while *higd1a* and *pir* exhibited delayed overexpression at 96 h, suggesting mitochondrial hypoxia and inflammation. Conclusions: Trichlorfon triggers a multifaceted toxic response characterized by initial activation of compensatory pathways (stress response, antioxidant defense, and anti-apoptotic mechanisms) followed by late-phase metabolic collapse, mitochondrial hypoxia, and inflammation, with both time- and dose-dependent effects. These findings demonstrate that even sublethal concentrations disrupt hepatic homeostasis and support the use of these genes as molecular biomarkers for environmental monitoring in aquaculture.

## Simple Summary

Chemical antiparasitic treatments are common in aquaculture but may harm the fish themselves. We investigated the effects of trichlorfon, an organophosphate pesticide, on tambaqui (*C. macropomum*). Juvenile fish were exposed to two sublethal concentrations for up to 96 h, and we analyzed the expression of genes related to stress (*fkbp5*), apoptosis (*p53*, *pim-2*), energy metabolism (*me1*, *bbox1*), and inflammation or hypoxia (*pir*, *higd1a*). Trichlorfon induced acute stress and genotoxic responses (*fkbp5*, *p53*), disrupted metabolic and antioxidant pathways (*me1*, *bbox1*), and triggered late inflammatory and hypoxic processes (*pir*, *higd1a*). These molecular alterations reveal that trichlorfon, even at low doses, can impair energy balance, promote inflammation, and endanger fish welfare—highlighting the need for safer parasite control strategies in aquaculture.

## 1. Introduction

The intensification of aquaculture in recent decades has led to a growing reliance on chemical substances for disease control, particularly for parasitic infections, which directly impact the zootechnical performance and survival of farmed fish. Among these substances, trichlorfon stands out as a widely used organophosphate due to its effectiveness as an antiparasitic agent. Several countries in Asia and Africa authorize its use, especially in agriculture and aquaculture. In Brazil, trichlorfon is legally permitted with no established maximum dosage limit. However, its frequent application in aquatic environments raises concerns regarding potential toxic effects, even at sublethal concentrations, on non-target organisms, including economically important species such as tambaqui (*Colossoma macropomum*) [1].

Tambaqui (*C. macropomum*) is one of the most widely farmed freshwater fish species globally and ranks as the second most cultivated species in Brazil, the largest producer of continental aquaculture in the Americas and the eighth largest worldwide [2]. The most recent available global production data for tambaqui in aquaculture dates back to 2016, when output reached approximately 142,000 metric tons. By 2022, Brazil alone accounted for 241,000 metric tons of tambaqui production [3]. The species has also expanded into Asian countries, notably China, Indonesia, Malaysia, Myanmar, and Vietnam [4]. In the Brazilian context, this native Amazonian species plays a vital role in the regional bioeconomy, serving as a key pillar of national aquaculture and generating significant socioeconomic benefits for traditional riverine communities [3,5].

Several studies have shown that trichlorfon can induce oxidative stress, modulate apoptotic pathways, and alter gene expression in various fish species. In carp, for example, trichlorfon exposure increased the activity of pro-oxidant enzymes such as xanthine oxidase, elevated lipid peroxidation levels (measured as MDA), and enhanced apoptosis rates in hepatocytes [6]. Similar findings in carp have also reported pronounced apoptotic effects and oxidative damage in gill tissues and erythrocytes, along with altered levels of glutathione and antioxidant enzymes such as superoxide dismutase (SOD), catalase (CAT), and glutathione peroxidase (GPx) [7].

Other authors have reported that trichlorfon also induces hematological alterations, including reductions in red blood cell count, hemoglobin levels, and plasma protein concentrations, as well as upregulation of stress-related genes such as *HSP70* and *CYP1A* [8]. Additionally, immunotoxic effects have been observed, with changes in the expression of immune-related genes such as *propor*, *LGBP*, and *SOD* in crustaceans exposed to trichlorfon, indicating significant impacts on immune system integrity [9].

From a molecular perspective, significant alterations in biomarker genes have also been identified in species such as *Pangasianodon hypophthalmus*, where trichlorfon exposure led to the induction of genes such as *HSP70*, *CYP1B*, and *COI*, along with suppression of *AChE* and *GH*. These findings highlight the potential of molecular biomarkers as a basis for environmental monitoring systems [10].

In tambaqui, Carvalho et al. (2024) [11] reported that trichlorfon exposure induced changes in the expression of the *GST* gene, which is associated with the antioxidant defense system, suggesting a potential reduction in biotransformation capacity in exposed fish. Significant alterations were also observed in the hepatic transcriptome, indicating that trichlorfon disrupts hundreds of metabolic pathways, including those related to neurotransmitter regulation, energy metabolism, and apoptosis [12].

In this context, the present study aimed to evaluate the effects of trichlorfon at sublethal concentrations based on the LC_50–96h_ values [13] on the expression of genes involved in stress response (*fkbp5*), apoptosis (*p53*, *pim-2*), energy metabolism (*me1*, *bbox1*), and inflammatory and hypoxic regulation (*pir*, *higd1a*) in juvenile tambaqui. Together, these molecular markers encompass key physiological processes—neuroendocrine activation, DNA damage response, redox balance, mitochondrial function, and inflammation—providing an integrated view of the cellular mechanisms underlying trichlorfon toxicity in fish. This multi-gene approach enhances the mechanistic understanding of organophosphate effects and strengthens the use of molecular biomarkers for environmental risk assessment in tropical aquaculture systems.

## 2. Materials and Methods

### 2.1. Ethics Statement

All experimental procedures were conducted in accordance with the guidelines of the Ethics Committee on Animal Experimentation of the Federal University of Amazonas (Manaus, AM, Brazil) and were approved under protocol number 030/2018.

### 2.2. Experimental Design

The specimens used in this study were obtained from the Experimental Farm of the Federal University of Amazonas, located on BR-174 Highway, km 38, Presidente Figueiredo access road, Manaus, AM, Brazil. This source was selected because there was no record of trichlorfon usage for parasite control in tambaqui at this location. The collected specimens, originating from the same batch, were transported to the Wet Laboratory of Parasitology, Morphology, and Fish Genetics at the Federal University of Amazonas, Manaus, AM, Brazil. The fish underwent a 60-day acclimation period in 310 L polyethylene tanks with continuous water circulation and aeration. During this period, the fish were fed a commercial growth feed containing 28% crude protein (Nutripiscis by Presence, Brasília, Brazil). Water quality parameters were monitored throughout the acclimation period using a multiparameter analyzer (pH/ORP, DO, EC, GPS; HI9829-10041, Hanna Instruments, Woonsocket, RI, USA). Temperature (°C), pH, and dissolved oxygen (mg/L) values were recorded at 25.34 ± 0.2 °C, 7.11, and 5.76 ± 1.1 mg/L, respectively.

The nominal concentrations of trichlorfon used for juvenile tambaqui exposure were based on the LC_50–96h_ value of 0.870 mg/L, as reported by Silva et al. (2020) [13]. The trichlorfon solution was prepared using the commercial formulation Masoten^®^ (Bayer S.A., Diegem, Belgium), which contains 80% trichlorfon and 20% solvent vehicle per 100 g of product. The compound was weighed according to the target concentrations and dissolved in distilled water.

**Figure 1:**
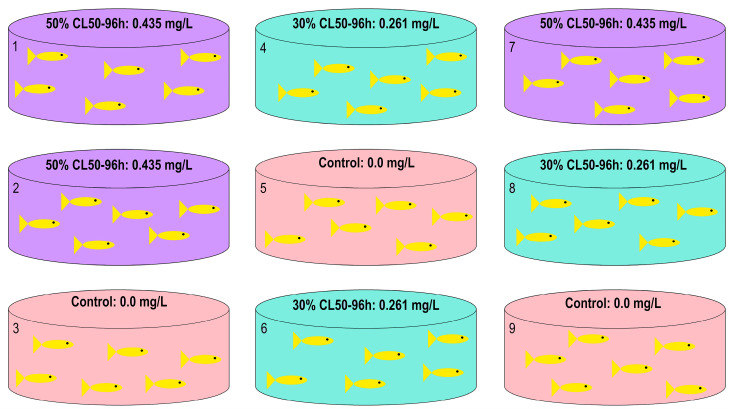
Experimental design for sampling tambaqui (*C. macropomum*) to obtain liver tissue samples.

Based on this, the fish were divided into three groups, each with three replicates: a control group (no trichlorfon exposure) and two treatment groups exposed to trichlorfon concentrations of 0.261 mg/L (30% of the LC_50–96h_) and 0.435 mg/L (50% of the LC_50–96h_). These concentrations were selected based on preliminary tests to ensure fish survival throughout the 96 h exposure period. Figure 1 provides an illustrative schematic of the experimental design described.

A total of 54 fish of the same age but with varying sizes were distributed across nine tanks, with six fish per tank. The average body weight was 16.45 ± 4.8 g and the average total length was 10.3 ± 0.99 cm. Prior to the start of the experiment, water circulation in the tanks was stopped and the water volume was adjusted to 60 L. Aeration was maintained throughout the 96 h exposure period, and fish were not fed during this time. Tank assignment to treatment groups, as well as the order of fish sampling, was randomized by drawing lots to ensure complete randomization of the experiment. Three sampling time points were established: 48, 72, and 96 h of exposure, each with a corresponding control group. Fish were euthanized by spinal cord concussion (according to Guidelines for the Euthanasia of Animals), and liver samples were immediately collected and preserved in Trizol Reagent^®^ (Invitrogen by Applied Biosystems, Woburn, MA, USA) for subsequent molecular analyses.

### 2.3. Quantitative Real-Time PCR (qPCR)

For the qPCR analysis, total RNA was extracted from liver samples using the Trizol Reagent^®^ protocol (Invitrogen by Applied Biosystems, Woburn, MA, USA), following the manufacturer’s instructions. RNA integrity was assessed by 1% denaturing agarose gel electrophoresis (1× MOPS buffer, formaldehyde, and agarose) with SYBR^®^ Safe Gel Satin (Invitrogen by Applied Biosystems, Eugene, OR, USA) and quantified using a NanoDrop 2000c spectrophotometer (Thermo Fisher Scientific, Wilmington, DE, USA). Prior to cDNA synthesis, samples were treated with Ambion™ DNase I (RNase-free) (Applied Biosystems by Thermo Fisher Scientific, Waltham, MA, USA) according to the manufacturer’s protocol to minimize the risk of genomic DNA contamination. Single-stranded cDNA was synthesized using the High-Capacity cDNA Reverse Transcription Kit (Applied Biosystems by Thermo Fisher Scientific, Waltham, MA, USA), following the manufacturer’s instructions, using 2000 ng of total RNA. The resulting cDNA was subsequently quantified with a FluorQuant™ Fluorometer (Loccus Biotechnology™, São Paulo, Brazil).

The entire qPCR assay was conducted following the guidelines described by Bustin et al. (2009) [14] in the Minimum Information for Publication of Quantitative Real-Time PCR Experiments (MIQE). Specific primers were designed for the target genes: FKBP prolyl isomerase 5 (*fkbp5*; NCBI Reference Sequence: XM_036570942.1), malic enzyme 1 (*me1*; NCBI Reference Sequence: XM_036555791.1), Pim-2 proto-oncogene, serine/threonine kinase (*pim-2*; NCBI Reference Sequence: XM_036579345.1), Tumor protein p53-inducible nuclear protein 2 (*p53*; NCBI Reference Sequence: XM_036580815.1), pirin (*pir*; NCBI Reference Sequence: XM_036563157.1), Gamma-butyrobetaine hydroxylase 1 (*bbox1*; NCBI Reference Sequence: XM_036584252.1) and HIG1 hypoxia inducible domain family member 1A (*higd1a*; NCBI Reference Sequence: XM_036597339.1). Table 1 provides a list of the primers used in this study. Two housekeeping genes, *gapdh* and 18S rDNA, previously validated by Nascimento et al. (2016) [15], were used for normalization. qPCR reactions were performed using the Amplio96™ Real-Time PCR System (Loccus Biotechnology^TM^, Brazil) and SYBR™ Green PCR Master Mix (Applied Biosystems by Thermo Fisher Scientific, Waltham, MA, USA), following the manufacturer’s instructions.

### 2.4. Statistical Analysis

The 2^−ΔΔCt^ method, as described by Livak and Schmittgen (2001) [16], was used to calculate relative gene expression levels. All statistical analyses were conducted using SigmaPlot version 15.0 and the AI Manus platform (https://manus.im/app, accessed on 9 December 2025).

For the genes *fkbp5*, *me1*, *pim-2*, *higd1a*, *bbox1*, and *p53*, a two-way analysis of variance (Two-Way ANOVA) was performed to simultaneously assess the main effects of Time (48 h, 72 h, and 96 h) and Dose (0%, 30% of the 96 h LC_50_, and 50% of the 96 h LC_50_), as well as the interaction between these factors on gene expression levels. The ANOVA was followed by tests for normality (Shapiro–Wilk) and homogeneity of variances (Brown–Forsythe). When significant differences were detected, multiple comparisons of means were conducted using Tukey’s test, with a significance level of α = 0.05.

For the *pir* gene, which did not meet the assumptions of normality or homogeneity, the nonparametric Mann–Whitney test was applied.

This approach allowed the identification of the main effects of Time, Dose, and their interaction (Time × Dose) on the expression of the evaluated genes, as well as the estimation of adjusted means and post hoc comparisons among the different factor levels and their combinations.

## 3. Results

The analysis of gene expression in the liver of juvenile tambaqui (*C. macropomum*) exposed to trichlorfon revealed a complex molecular response, modulated by both dose and exposure time. The Two-Way Analysis of Variance (Two-Way ANOVA) indicated a significant interaction between dose and time factors for most of the genes evaluated: *fkbp5* (F(4,18) = 10,218; *p* < 0.001), *p53* (F(1,8) = 315,363; *p* < 0.001), *pim-2* (F(4,18) = 28,4717; *p* < 0.001), *me1* (F(4,18) = 5,605; *p* = 0.004), *bbox1* (F(4,18) = 46,170; *p* < 0.001) e *higd1a* (F(4,18) = 13,690; *p* < 0.001). This interaction justifies the analysis of dose effects within each exposure time.

### 3.1. Genes Related to Stress, Apoptosis, and Inflammation

Exposure to trichlorfon induced significant alterations in the expression of genes associated with stress, apoptosis, and inflammatory responses in the liver of juvenile tambaqui (*C. macropomum*). The genes *fkbp5*, *pim-2*, *p53*, and *pir* exhibited distinct expression patterns over time and across the tested concentrations, reflecting a multifaceted molecular cascade in response to the xenobiotic compound. These genes showed distinct temporal responses, characterized by an early activation phase (48 h) followed by a late inflammatory response (96 h).

**Figure 2:**
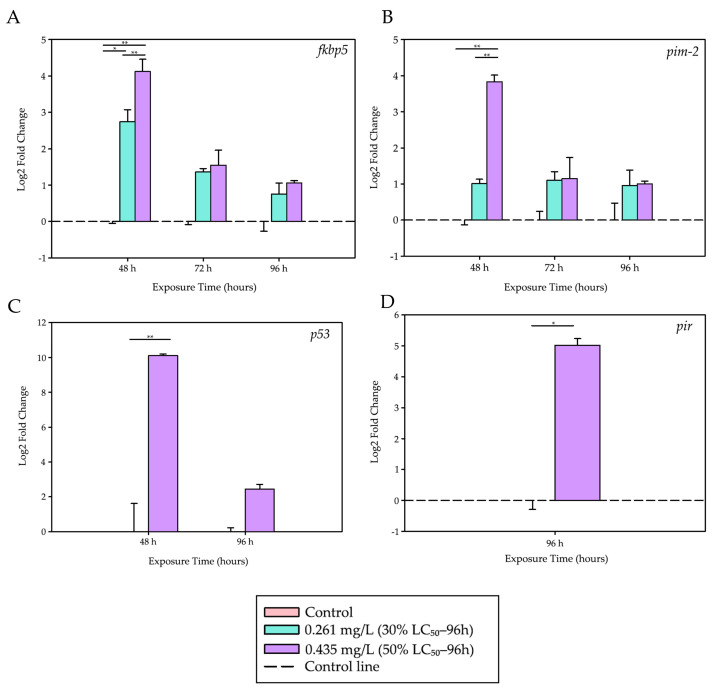
Differential expression levels of stress-, apoptosis-, and inflammation-related genes. Stress, apoptosis, and inflammation responses induced by trichlorfon in the liver of tambaqui (*C. macropomum*). (**A**) *fkbp5*, (**B**) *pim-2*, (**C**) *p53*, and (**D**) *pir*. Relative expression levels (Log_2_ Fold Change) are shown for different concentrations (Control, 30% LC_50–96 h_—0.261 mg/L, 50% LC_50–96 h_—0.435 mg/L) and exposure times (48 h, 72 h, and 96 h). For each analysis, 4 biological replicates and 3 technical replicates were used. Lines represent statistical differences: * *p* < 0.05 and ** *p* < 0.01; *fkbp5*, *pim-2* and *p53*—Two-Way ANOVA; *pir*—Mann–Whitney Test.

Table 2 presents the biological rationale for the grouping of these genes, based on their physiological functions and the temporal patterns observed in this study. Figure 2 shows the graphs with the Log_2_ Fold Change values calculated for these genes.

After 48 h of exposure, the *fkbp5* gene, a marker of acute stress response, showed a significant upregulation at both trichlorfon concentrations compared to the control group (*p* = 0.022 for 0.261 mg/L; *p* < 0.001 for 0.435 mg/L). The response was dose-dependent, with expression at 0.435 mg/L being significantly higher than that observed at 0.261 mg/L (*p* < 0.001).

During the same period, the pro-apoptotic gene *p53* was strongly induced at the highest concentration (0.435 mg/L) compared to the control (*p* < 0.001). Similarly, the anti-apoptotic gene *pim-2* was also upregulated at 0.435 mg/L relative to both the control group (*p* < 0.001) and the lower concentration (0.261 mg/L; *p* < 0.001), with a 14.3-fold increase. At 72 and 96 h, no significant differences were observed in the expression levels of *fkbp5* and *pim-2* among the treatment groups.

A late inflammatory response was observed at 96 h, with the *pir* gene showing an approximately 32-fold increase in expression in the group exposed to 0.435 mg/L compared to the control (t = −7.172; df = 4; *p* = 0.002), suggesting a possible activation of inflammatory pathways at later stages of exposure.

Table 3 presents a summary of the quantitative results for these genes over time and across the evaluated concentrations.

### 3.2. Genes Related to Energy Metabolism and Hypoxia

Exposure to trichlorfon also affected genes associated with energy metabolism and the mitochondrial response to hypoxia. The genes *me1*, *bbox1*, and *higd1a* exhibited biphasic expression patterns, reflecting a progressive collapse of redox metabolism and energy production. These genes exhibited significant alterations during both the early and late phases of trichlorfon exposure.

**Figure 3:**
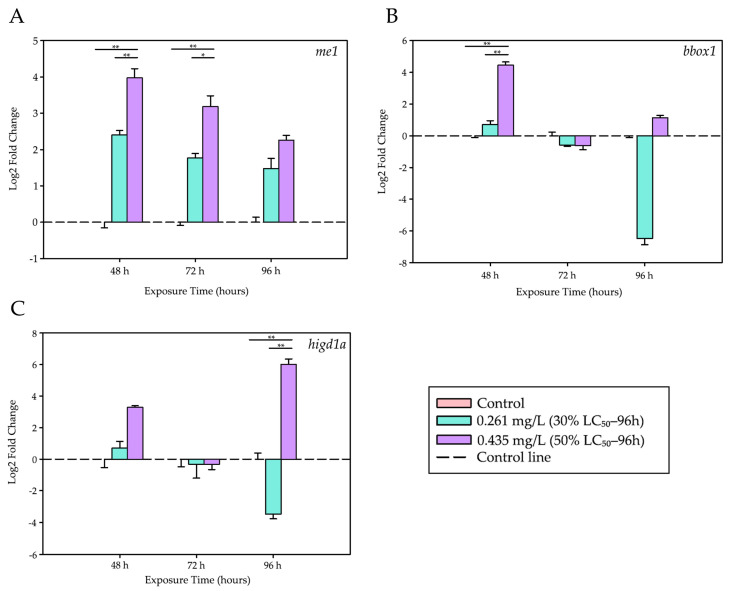
Differential expression levels of energy metabolism- and hypoxia-related genes. Modulation of genes associated with energy metabolism and mitochondrial hypoxia in the liver of tambaqui (*C. macropomum*) exposed to trichlorfon. (**A**) *me1*, (**B**) *bbox1*, and (**C**) *higd1a*. Relative expression levels (Log_2_ Fold Change) are shown for different concentrations (Control, 30% LC_50–96 h_—0.261 mg/L, 50% LC_50–96 h_—0.435 mg/L) and exposure times (48 h, 72 h, and 96 h). For each analysis, 4 biological replicates and 3 technical replicates were used. Lines represent statistical differences: * *p* < 0.05 and ** *p* < 0.01; Two-Way ANOVA.

Table 4 presents the biological rationale for the grouping of these genes, and Figure 3 shows the graphs with the Log_2_ Fold Change values calculated for these genes.

After 48 h, exposure to 0.435 mg/L of trichlorfon resulted in a marked increase in the expression of the *me1* gene (associated with redox metabolism) and the *bbox1* gene (related to lipid metabolism). Both genes showed significantly higher expression levels compared to the control group (*p* < 0.001) and the lower concentration (0.261 mg/L; *p* < 0.001).

At 72 h, only *me1* maintained elevated expression levels in the 0.435 mg/L group when compared with the control (*p* < 0.001) and the lower concentration (*p* = 0.009).

Finally, at 96 h, a pronounced induction of the *higd1a* gene, a marker of mitochondrial hypoxia, was observed. Its expression was significantly higher in the group exposed to 0.435 mg/L than in both the control group (*p* < 0.001) and the 0.261 mg/L group (*p* < 0.001). During this same period, *me1* and *bbox1* did not show significant differences among treatments.

Table 5 summarizes the main quantitative results observed for these genes.

## 4. Discussion

The results of this study demonstrate that the antiparasitic trichlorfon, even at sublethal concentrations, exerts significant effects on the expression of key hepatic genes in juvenile tambaqui (*C. macropomum*). The expression patterns of *fkbp5*, *me1*, and *pim-2* suggest physiological stress responses, disruptions in energy metabolism, and potential impairment of cell proliferation, consistent with transcriptomic findings reported by Silva et al. (2025) [12]. These results reinforce the concern that trichlorfon, despite its widespread use in aquaculture, can disrupt molecular homeostasis in exposed organisms, even over short-term exposures.

The *fkbp5* gene showed a marked increase in expression within the first 48 h of exposure, particularly at the highest concentration tested (0.435 mg/L), with an upregulation of up to 18.3-fold. This gene is associated with glucocorticoid-mediated stress responses and is a known marker of activation of the hypothalamus–pituitary–interrenal (HPI) axis in teleost fish. Its regulation by environmental contaminants has been previously reported. Notably, studies have shown *fkbp5* overexpression in zebrafish (*Danio rerio*) exposed to environmental stressors such as SSRIs, highlighting its sensitivity as an HPI activation marker [17]. In fish, HPI axis activation triggers cortisol release, which in turn induces expression of stress-related genes like *fkbp5*, contributing to the maintenance of cellular homeostasis under adverse conditions.

Regarding *me1*, we observed up to a 16-fold increase in gene expression at both 0.261 mg/L (30% LC_50–96 h_) and 0.435 mg/L (50% LC_50–96 h_) after 48 and 72 h of exposure. This effect persisted at 96 h only in the 50% treatment group (9.5-fold increase). The *me1* gene encodes the malic enzyme 1 (ME1), which plays a central role in NADPH production, a crucial cofactor for the regeneration of reduced glutathione (GSH), a key component of the cellular antioxidant defense system. The sustained upregulation of me1 suggests a compensatory attempt to maintain NADPH synthesis and antioxidant capacity. However, despite this transcriptional response, the cellular antioxidant defense may still be insufficient to fully counteract oxidative stress, potentially resulting in reactive oxygen species (ROS) accumulation and subsequent cellular damage.

This observation aligns with findings by Duncan et al. (2020) [18], who reported no changes in glutathione S-transferase (GST) activity in juvenile tambaqui exposed to the same compound and duration. Similarly, Carvalho et al. (2024) [11] observed no changes in *gst* gene expression under equivalent conditions. Taken together, these results suggest a failure to activate antioxidant defense mechanisms, implying that trichlorfon may not stimulate, and may even suppress, compensatory antioxidant pathways over time. Since ME1 supplies the NADPH required for GST activity, its downregulation may represent a critical metabolic bottleneck: even if GST is present and functional, its activity may be limited by cofactor availability.

This pattern contrasts with results from Souza et al. (2021) [19], who reported rapid and significant activation of antioxidant enzymes such as SOD and GST in tambaqui exposed to malathion. That early response was interpreted as a compensatory mechanism to counteract free radicals generated by initial xenobiotic exposure. The discrepancy may reflect intrinsic differences between the organophosphates (malathion vs. trichlorfon) or exposure timing and the functional status of the antioxidant system (e.g., activation vs. suppression).

While malathion appears to trigger an effective early antioxidant response, our data indicate that trichlorfon may impair this response at later stages, particularly the NADPH-ME1 pathway. It is also possible that alternative NADPH-generating pathways, such as glucose-6-phosphate dehydrogenase (G6PDH), are being prioritized under these conditions, though apparently insufficient to fully restore redox homeostasis.

Pim-2 kinase is involved in cell proliferation and survival under stress by regulating apoptosis. In this study, *pim-2* expression was significantly increased, especially in the group exposed to the higher trichlorfon concentration (14.3-fold increase at 48 h). This early change suggests a strong activation of molecular pathways associated with cellular survival and stress resistance. Given that PIM-2 functions as a negative regulator of apoptosis, this upregulation may represent an adaptive response to protect cells from trichlorfon-induced stress. A 2.6-fold increase in *pim-2* expression was also observed in the 50% group after 72 h, and a 2.2-fold increase in the 30% group, indicating persistent activation at both concentrations. This response supports the hypothesis that trichlorfon triggers compensatory protective cellular mechanisms, with *pim-2* acting as a key survival factor during the acute phase of toxicity, alongside activation of antioxidant pathways (via *me1*).

These findings are consistent with Cericato et al. (2008) [20], who reported impaired stress responses in jundiá (*Rhamdia quelen*) exposed to pesticides, suggesting loss of physiological homeostasis. Although *pim-2* was not directly assessed, such endocrine and physiological disruptions may reflect similar impairments, where early *pim-2* suppression compromises cellular resistance systems. Additionally, our findings align with those of Baldissera et al. (2019) [21], who reported energy homeostasis disturbances and oxidative damage in jundiá exposed to trichlorfon. The upregulation of *pim-2*, a gene linked to cell survival, may reflect an initial compensatory activation of cytoprotective mechanisms, increasing fish susceptibility to oxidative stress and apoptosis.

Although direct assessments of *pim-2* expression in fish exposed to organophosphates are lacking, our findings are consistent with Marín Méndez et al. (2014) [22], who reported severe histological damage in pirapitinga (*Piaractus brachypomus*) exposed to trichlorfon, indicating impaired cellular homeostasis. Additionally, studies in other biological models have shown that genes such as *bax*, *bcl-2*, *p53*, and *PUMA* play central roles in regulating apoptosis in response to oxidative stress [23], suggesting that *pim-2* may function within this regulatory network. Its early upregulation may reflect activation of protective mechanisms, leading to greater vulnerability to apoptosis in sensitive tissues such as liver and gills.

When considered alongside the reduced *me1* expression and findings from Duncan et al. (2020) [18] and Carvalho et al. (2024) [11], which reported no changes in *gst* activity or expression in trichlorfon-exposed tambaqui, a consistent pattern emerges: while some cytoprotective pathways are activated (*me1*, *pim-2*), others remain unresponsive (*gst*). This suggests a deficient cellular response to chemical stress, increasing the potential for oxidative damage and apoptosis.

In contrast, Souza et al. (2023) [24] showed that malathion exposure induced increased expression of pro-apoptotic genes (*tp53*, *bax*) and decreased expression of the anti-apoptotic gene *bcl-2* in tambaqui, indicating activation of the apoptotic pathway. In the present study, however, the data suggest a distinct mechanism for trichlorfon toxicity, in which both pro-apoptotic (*p53*) and anti-apoptotic (*pim-2*) pathways are simultaneously activated during the acute phase, suggesting a complex cellular response where survival and death signals coexist. These differences underscore the diversity of toxicological mechanisms among organophosphates and highlight the need for more in-depth comparative studies on their molecular effects in aquatic organisms.

Interestingly, we observed a trend toward partial recovery of gene expression levels at 96 h, especially for *pim-2* and *me1*, suggesting a possible physiological adaptation to trichlorfon exposure. This partial recovery may reflect the onset of compensatory mechanisms over time, although it appears insufficient to fully restore cellular homeostasis, as evidenced by the continued elevation of *me1* at 50% LC_50_ even after 96 h.

In addition to the responses observed in *fkbp5*, *me1*, and *pim-2*, the expression of *higd1a* (hypoxia-inducible gene domain family member 1A) revealed one of the most informative patterns in this study. This gene is a well-known regulator of cytochrome c oxidase (CcO) activity, the terminal complex of the mitochondrial respiratory chain, and plays a crucial role in cellular adaptation to hypoxia and oxidative stress [25,26]. The initial 8.7-fold induction at 48 h in the higher concentration suggests an acute compensatory response aimed at optimizing oxidative phosphorylation efficiency under chemical stress. However, the return to baseline levels at 72 h, followed by a massive 724.5-fold induction at 96 h, indicates a second wave of response, likely associated with persistent cellular hypoxia, as observed in other models [27]. In contrast, the 1.3-fold decrease observed at the lower concentration after 96 h suggests a collapse of this adaptive capacity, possibly a hormetic effect, in which lower doses can be more deleterious in the long term for certain metabolic pathways [28].

The *pir* (pirin) gene is a redox stress sensor that interacts with the transcription factor *NF-κB*, a master regulator of inflammatory and apoptotic responses [29,30]. The absence of detectable expression at 48 and 72 h, followed by a strong 31.8-fold induction at 96 h in the higher concentration, characterizes *pir* as a biomarker of late stress. This delayed induction may signal a transition from an acute cellular stress response to a chronic inflammatory phase, which may ultimately lead to apoptosis.

The upregulation of the *me1* gene, involved in NADPH production, was accompanied by drastic alterations in the *bbox1* (gamma-butyrobetaine dioxygenase) gene, which encodes the enzyme that catalyzes the final step in the biosynthesis of L-carnitine, a molecule essential for the transport of fatty acids into the mitochondria for β-oxidation [31]. The substantial 22.23-fold increase observed at 48 h in the higher concentration provides strong evidence of severe trichlorfon-induced genotoxicity. The subsequent decrease in expression at 72 and 96 h may indicate the initiation of a repair process or the elimination of severely damaged cells through apoptosis.

The *p53* gene is universally known as the “guardian of the genome,” being activated in response to DNA damage to orchestrate cell cycle arrest, DNA repair, or apoptosis [32]. The massive 332.2-fold induction observed at 48 h in the higher concentration provides the strongest evidence of severe trichlorfon-induced genotoxicity. This level of activation represents an extreme cellular alarm signal, indicating that DNA damage has exceeded the basal repair capacity, corroborating previous studies demonstrating the genotoxic potential of organophosphates in fish [33]. The subsequent decrease in expression at 72 and 96 h may indicate the initiation of a repair process or the elimination of severely damaged cells through apoptosis. Notably, the absence of detectable p53 expression at 48 h in the 30% group suggests a threshold effect, where only concentrations above a certain level trigger the DNA damage response mediated by p53, consistent with the findings of Souza et al., 2023 [24], who reported a dose-dependent activation of *tp53* in tambaqui exposed to malathion.

The integrated analysis of the seven evaluated genes provides a comprehensive understanding of the molecular mechanisms triggered by trichlorfon exposure in *C. macropomum* (tambaqui). The results reveal a triphasic toxicity response characterized by successive events of cellular stress, metabolic imbalance, and late inflammation.

In the acute phase (48 h), activation of genes associated with neuroendocrine and genotoxic stress, such as *fkbp5* and *p53*, was observed, reflecting induction of the HPI axis and the response to DNA damage. Simultaneously, an initial compensatory response in energy metabolism occurred, evidenced by the strong induction of *bbox1*, indicating an attempt to maintain mitochondrial and oxidative homeostasis under early toxicity.

During the transitional phase (72 h), sustained elevation of genes related to cell survival and redox metabolism, such as *pim-2* and *me1*, was observed, suggesting collapse of energy and antioxidant defense pathways. Finally, in the late phase (96 h), reactivation of mitochondrial and inflammatory genes, such as *higd1a* and *pir*, became prominent, indicating conditions of hypoxia and persistent oxidative stress accompanied by secondary inflammation.

Taken together, these results demonstrate that even at sublethal concentrations, trichlorfon triggers a complex molecular response characterized by initial activation of compensatory pathways (*pim-2*, *me1*, *bbox1*) followed by late-phase metabolic collapse (*bbox1* reduction, *higd1a* and *pir* induction), ultimately in the liver of tambaqui (*C. macropomum*), affecting energy production, redox balance, and cellular integrity. The integration of these findings reinforces the role of these genes as sensitive biomarkers of chemical stress, useful for monitoring organophosphate pesticides in aquatic environments.

## 5. Conclusions

Trichlorfon, even at sublethal concentrations, elicited complex molecular alterations in the liver of tambaqui (*C. macropomum*), affecting stress, metabolic, and apoptotic pathways in a time- and dose-dependent manner. These findings emphasize the potential risk of organophosphate use in aquaculture, as it may compromise fish welfare and productivity even under conditions considered safe. The identification of responsive genes associated with stress, energy metabolism, and hypoxia supports their applicability as sensitive molecular biomarkers for environmental monitoring. Although this study focused on acute exposure, the results provide a mechanistic basis for understanding trichlorfon-induced toxicity and highlight the need for further investigations on chronic exposure and interactions with other environmental stressors. Overall, this work reinforces the importance of integrating molecular tools into aquaculture management to promote safer and more sustainable practices.

## Figures and Tables

**Table 1:** Primers designed for the qPCR reaction.

Gene	Sequence		Tm (°C)	Amplicon	Efficiency
*fkbp5*	Forward	5′ ccaaaggagacgatccgaatac 3′	62.9	85	102%
	Reverse	5′ tgtctgtaggaatgccgtttag 3′	62.8		
*me1*	Forward	5′ catccatgaccgctctcatatc 3′	62.9	107	98%
	Reverse	5′ gatcacccaatcccagaatcc 3′	62.9		
*pim-2*	Forward	5′ cagagtttacagtccaccagag 3′	62.5	103	92%
	Reverse	5′ cccacagaccatgtcaaaga 3′	62.6		
*p53*	Forward	5′ ggaaggtgagagcgagaataaa 3′	62.6	119	91%
	Reverse	5′ gctgcttagtgggctacaata 3′	62.7		
*pir*	Forward	5′ tctacactctctcgggattctt 3′	62	104	95%
	Reverse	5′ cactctgacacagtcaccatc 3′	62		
*bbox1*	Forward	5′ gtggttcagtaggtgtctaagc 3′	62.8	99	95%
	Reverse	5′ gaggtgaagggttgtcactaaa 3′	62.8		
*higd1a*	Forward	5′ gaagcagagagggaacatgaa 3′	62	106	98%
	Reverse	5′ catggagtagaccacacctaag 3′	62		

The table presents the primers designed for the selected genes *fkbp5*, *me1*, *pim-2*, *p53*, *pir*, *bbox1* and *higd1a.* The sequences were obtained from the *C. macropomum* reference genome.

**Table 2:** Biological rationale for the grouping of stress-, apoptosis-, and inflammation-related genes analyzed in the liver of tambaqui (*C. macropomum*) exposed to trichlorfon.

Gene	Main Function	Observed Temporal Role	Function in the Physiological and Toxicological Context
*fkbp5*	Stress response via the HPI axis	Acute induction at 48 h	Indicates initial activation of the stress response and neuroendocrine axis
*p53*	Regulation of DNA damage and apoptosis	Massive induction at 48 h	Signals genotoxicity and early apoptosis in hepatocytes
*pim-2*	Regulation of cell survival	Early suppression at 48 h and 72 h	Indicates failure of cellular protective mechanisms
*pir*	Redox sensor and modulator of NF-κB	Late induction at 96 h	Indicates inflammatory activation and secondary oxidative stress

**Table 3:** Summary of relative expression levels of stress-, apoptosis-, and inflammation-related genes in the liver of tambaqui (*C. macropomum*) exposed to trichlorfon.

Genes	Exposure Time/Concentration						
	48 h		72 h		96 h		General Trend
	30% LC_50–96 h_	50% LC_50–96 h_	30% LC_50–96 h_	50% LC_50–96 h_	30% LC_50–96 h_	50% LC_50–96 h_	
*fkbp5*	↑ 7.1x *	↑ 18.3x *	↑ 2.6x	↑ 3.2x	↑ 1.7x	↑ 2x	Acute initial stress; partial late adaptation
*p53*	<LOD	↑ 332.2x *	<LOD	<LOD	<LOD	↑ 5.6x	Early genotoxic and apoptotic response
*pim-2*	↑ 2x	↑ 14.3x *	↑ 2.2x	↑ 2.6x	↑ 1.9x	↑ 1.8x	Suppression of cellular survival pathways
*pir*	<LOD	<LOD	<LOD	<LOD	<LOD	↑ 31.8 *	Late inflammatory induction

* *p* < 0.005; <LOD = below the limit of detection. ↑ Increase in gene expression levels.

**Table 4:** Biological rationale for the grouping of energy metabolism– and oxidative metabolism–related genes analyzed in the liver of tambaqui (*C. macropomum*) exposed to trichlorfon.

Gene	Main Function	Observed Temporal Role	Function in the Physiological and Toxicological Context
*me1*	Production of NADPH and cellular redox balance	Suppression between 48 and 72 h	Indicates energy deficit and reduction in antioxidant defense
*bbox1*	Synthesis of L-carnitine and β-oxidation	Induction at 48 h; collapse at 96 h	Indicates an initial compensatory response followed by metabolic failure
*higd1a*	Regulation of the mitochondrial respiratory chain under hypoxia	Biphasic induction, peak at 96 h	Indicates mitochondrial adaptation to prolonged oxidative stress

**Table 5:** Summary of relative expression levels of energy metabolism- and hypoxia-related genes in the liver of tambaqui (*C. macropomum*) exposed to trichlorfon.

Genes	Exposure Time/Concentration						
	48 h		72 h		96 h		General Trend
	30% LC_50–96 h_	50% LC_50–96 h_	30% LC_50–96 h_	50% LC_50–96 h_	30% LC_50–96 h_	50% LC_50–96 h_	
*me1*	↑ 5.3x	↑ 16x *	↑ 3.4x	↑ 9.5x *	↑ 2.9x	↑ 4.8x	Initial metabolic suppression with slight late recovery
*bbox1*	↑ 1.67x	↑ 22.23 *	↓ 1.5x	↓ 1.5x	↓ 82.2x	↑ 2.21x	Biphasic response: initial compensation followed by collapse
*higd1a*	↑ 1.6x	↑ 8.7x	=	↓ 1.3x	↓ 11.3x	↑ 724.5x *	Mitochondrial adaptation to hypoxia and oxidative stress

* *p* < 0.005; <LOD = below the limit of detection. ↑↓ Increase or decrease in gene expression levels.

## Data Availability

The original contributions presented in this study are included in the article. Further inquiries can be directed to the corresponding author.
